# From Inter-Racial Solidarity to Action: Minority Linked Fate and African American, Latina/o, and Asian American Political Participation

**DOI:** 10.1007/s11109-021-09750-6

**Published:** 2021-09-25

**Authors:** Nathan Kar Ming Chan, Francisco Jasso

**Affiliations:** University of California Irvine, Irvine, USA

**Keywords:** Minority linked fate, Political behavior, Identity politics, Political participation, Race, ethnicity, and politics

## Abstract

Recent literature in race, ethnicity, and politics has assessed how minority linked fate, defined as “the idea that ethnoracial minorities might share a sense of commonality that extends beyond their particular ethnoracial group to other ethnoracial groups (Gershon et al., in Politics Groups Identities 7(3):642–653, 2019),” shapes attitudes toward descriptive representation and support for coalition building. However, scholarship has yet to examine the influence of minority linked fate on political participation. We argue that similar to those who view the interests of co-ethnics as a proxy for their individual interests, Latina/os, Asian Americans, and African Americans who express linked fate with a more expansive minority community are more likely to take political action. This political participation results from senses of obligation to and solidarity with other racial minorities outside of their own. Results from the 2016 Collaborative Multiracial Post-Election Survey show that controlling for conventional measures of linked fate, minority linked fate is associated primarily with more system-challenging modes of political activity for Latina/os, Asian Americans, and African Americans. We conclude by positioning minority linked fate as a complementary heuristic to traditional notions of intra-racial linked fate and note how shared inter-racial linked fate informs our understanding of recent political activism among people of color.

**Supplementary Information:**

The online version contains supplementary material available at 10.1007/s11109-021-09750-6.

## Introduction

In 2020, the Black Lives Matter movement galvanized racial minorities to come together out of a sense of solidarity. However, inter-group solidarity across minority groups is not a new development. In the 1960s and 1970s, Latina/o, Asian, and African Americans led social movements articulating their demands on the political system in terms of solidarity with one another, expressing a shared experience as “people of color” (Pan, 2020; Perez, 2020). Individual racial groups have supported one another from a sense of interconnectedness that sees their individual group’s struggle as related to the challenges of other racial minorities.

Literature in race, ethnicity, and politics has primarily focused on how within-group racial solidarity^1^ via intra-racial linked fate, influences minorities’ political behavior. Yet, Latina/o, Asian Americans, and African Americans share commonalities in relation to a dominant White racial group. Moving scholarship in identity and political behavior forward, we ask: What impact does a sense of inter-racial minority linked fate have on the political participation of Latina/o, Asian Americans, and African Americans? How do such perceptions of across-group linked fate motivate more political action among people of color? In the following sections, we review the literature on intra-racial linked fate and theorize about minority linked fate as a complementary group-based resource for political behavior among marginalized groups. We then present evidence from the 2016 Collaborative Multiracial Post-Election Survey (CMPS), showing that Latina/os, Asian Americans, and African Americans who hold a stronger sense of minority linked fate are more likely to participate in politics, particularly system-challenging modes of political action. We conclude by discussing how minority linked fate informs our understanding of current political movements spearheaded by people of color.

## The Intra-racial Group Basis of Political Behavior

Scholars have long examined the determinants of electoral and non-electoral participation. Individual-level explanations have centered the importance of socioeconomic resources such as education and income (Verba & Nie, 1972; Verba et al., 1995) as well as psychological resources such as efficacy, interest in politics, and trust in government (Aldrich, 1993; Campbell et al., 1960). Verba et al.’s (1995) Civic Voluntarism Model, for example, synthesized individual-level accounts with institutional factors, having found that those who are recruited in politics respond with more political action (*see also* Rosenstone & Hansen, 1993). Individuals become active in political life based on whether one has the capacity, wants to do so, or is asked to participate.

However, it is important to note that scholars have emphasized racial group-specific explanations. For example, Dawson (1994) introduced the concept of linked fate, defined as the extent to which African Americans perceive their individual life circumstances are connected to the fate of others within their racial group. Under this framework, rationally procuring group interests will maximize one’s own self-interests as well. This “Black Utility Heuristic” derives from African Americans’ shared history of oppression and group devaluation. To the degree that race affects life experiences, Dawson (1994) posits that this racial in-group indicator is a meaningful construct for how African Americans navigate politics. The trajectory of this research has been supported by scholars who have explored group-based participation, emphasizing the importance of attachment toward racial in-group members (Miller et al., 1981; Shingles, 1981; Verba & Nie, 1972). Some have found insignificant or waning effects of group solidarity (Leighley & Vedlitz, 1999; Tate, 1991) while others argue the influence of group solidarity depends on the specific measurements and modes of action being assessed (Chong & Rogers, 2005).

Group-based resources have further been used to explain the political behavior of non-Black minorities.^2^ Latina/os and Asian Americans do feel a sense of linked fate (Bowler & Segura, 2011; Masuoka & Junn, 2013; Sanchez & Masuoka, 2010; Wong et al., 2005, 2011), although this varies by generation and national origin (Masuoka, 2006; Sanchez & Masuoka, 2010), and is weaker and more malleable compared to African Americans (Chong & Kim, 2006; Junn & Masuoka, 2008; Masuoka, 2008; Masuoka & Junn, 2013). For non-Black minorities, within-group solidarity has yielded both positive (Sanchez, 2006; Valdez, 2011) and mixed results on political participation (Leighley & Vedlitz, 1999; Lien, 1994; Wong et al., 2005).

In commemorating Dawson’s (1994) contribution of linked fate to the political behavior literature, scholars revisited the impact of and confirmed the contemporary relevance of this heuristic on various racial groups (Marsh & Ramirez, 2019; Sanchez et al., 2019; Shaw et al., 2019). Furthermore, Gershon et al. (2019) advance the concept of a “minority linked fate,” which they define as “the idea that ethnoracial minorities might share a sense of commonality that extends beyond their particular ethnoracial group to other ethnoracial groups” (pp. 642–643). Their work here is an extension to Dawson’s (1994) seminal conceptualization of intra-racial linked fate.

The goal of this paper is to further build on political behavior research regarding group-based resources and minority linked fate. We do so by addressing an increasingly salient social identity defined by a *minority* and *non-minority* status. We theorize about how this minority status is politically relevant, particularly in the context of 2016 and the contemporary political moment. In addition, we theorize about why a shared minority linked fate mobilizes political participation specifically among people of color. Following this, we present data from the 2016 Collaborative Multiracial Post-Election Survey; we find that inter-racial minority linked fate is a meaningful heuristic for political activism among Asian, Latina/o, and African Americans that complements the insight gained from intra-racial linked fate. Our findings contribute to our understanding of how minorities can bring to bear multiple group-based resources on their decision to be involved in politics.

## Intra-Racial and Inter-Racial Group Solidarity

Seeing intra-racial linked fate alongside inter-racial linked fate requires establishing the grounds from which to expect that minority individuals can feel a sense of connectedness and common fate with those outside their individual racial in-groups. Identities are dynamic, subject to change, and flexible based upon circumstances and environment (Junn & Masuoka, 2008; Lopez & Espiritu, 1990). Further, individuals can hold multiple social categorizations as part of their self-concept and recategorize according to context (Barvosa, 2008; Gaertner et al., 1993, 2000; Garcia-Rios et al., 2019; Turner et al., 1987; Ufkes et al., 2012). As such, racial group identity is not mutually exclusive with a superordinate minority identity. Individuals can hold attachments toward their own racial in-group while viewing it as nested under a minority in-group that encompasses other racial groups (Perez, 2020).

Recently changing and evolving political contexts have made the divide between racial minorities and non-racial minorities particularly salient, compelling individuals of the former to feel similarly targeted through a common minority in-group identity. Accordingly, we note the incremental value in examining the consequences of inter-racial group solidarity, via minority linked fate, as a complement to intra-racial linked fate.

Politicians and elites play important roles in shaping the salience and awareness of group identities (Campbell et al., 1960; Perez, 2015; Rothschild, 2020; Zamora, 2011). Black elites, for example, have long used broad categories such as ‘people of color’ to express commonality between African Americans, Latina/os, and Asian Americans. In recent circumstances, Perez (2021b) notes that many identify themselves in these terms. Donald Trump’s negative rhetoric has reminded racial minority groups of their marginalized status in society (Perez, 2021a) and has pushed these individuals to consider not only standing in solidarity with others from within one’s own racial in-group but with other marginalized minorities as well.

Trump began his 2016 electoral bid with negative racial rhetoric. As early as 2011, he questioned President Obama’s birthplace and subsequently incited fear by connecting him to Muslims and ISIS. He also infamously declared racist remarks against Mexican immigrants when formally announcing his candidacy for president. His foreign policy attitudes towards China (Nguyen, 2017) and his interrupting of an Asian American student to ask if he was South Korean (Khalid, 2015) reflect long standing stereotypes of Asian Americans as foreign (Kim, 1999). These attacks on minorities were further brought to light as Trump failed to denounce White supremacists in Charlottesville and emboldened racist individuals to act upon their preexisting prejudices (Newman et al., 2019). More recently, Trump’s framing of the Covid-19 pandemic in racialized terms linked Asian Americans to the virus, sparking anti-Asian violence in record numbers across the United States (Griffin et al., 2019; Pan, 2020; Reny & Barreto, 2020; Chan et al., 2021; Jeung et al., 2021).

Trump’s threats were racial group-specific, but as no historically disadvantaged group has been left unscathed, such circumstances have pushed minorities to stand not only with their own racial group but with an overarching minority in-group. Trump’s use of negative racial rhetoric throughout his campaign and presidency has coincided with the growing use of common in-group identifiers, such as BIPOC (Black, Indigenous, and People of Color), which activists used prominently in the context of the 2020 Black Lives Matter protests (Garcia, 2020). The nature of these common in-group identifiers recognizes minority groups' shared non-White status in a way that is cautious not to erase each individual group’s uniqueness (see Beltrán, 2010; Sexton, 2008). Ultimately, this inter-group solidarity reinforces a sense of shared marginalization and need for collective action to improve group outcomes (Gaertner et al., 2000). Given the political climate after 2016 as characterized by Trump’s negative rhetoric, the relevance of one’s intra-racial in-group may have heightened, but an additional awareness of being a part of a broader racial minority community may have also further increased in political salience.

The intergroup relations literature has examined the conditions under which minorities can find shared experiences and come together in solidarity with each other. It is then sensible that Asian Americans and Latina/os with greater senses of intra-group considerations have a stronger sense of commonality with and support for African Americans (Kaufmann, 2003; Merseth, 2018; Nicholson et al., 2020; Sanchez, 2008). Perceptions of discrimination among Asian Americans and Latina/os are also related to a sense of commonality with African Americans (Craig & Richeson, 2012; Nicholson et al., 2020; Sanchez, 2008). Perceiving commonality with other racial groups additionally increases support for race-centered social movements. Among Asian Americans, inter-racial and intra-racial linked fate are associated with more support for Black Lives Matter (Merseth, 2018; *see also* Dowe et al., 2018). Additionally, Asian Americans who perceive commonality with Latina/os and African Americans are more likely to support a pathway to citizenship for undocumented immigrants, which Samson (2014) suggests may reflect a long standing Asian American civil rights agenda whose scope extends beyond their immediate panethnic in-group.

This literature suggests a record of minority groups coming together with those outside of their racial in-group. Recent circumstances may have also incentivized Latina/o, Black, and Asian Americans to stand together as one minority community. Racial minorities in the Trump era may continually draw identity boundaries within their own individual racial group, but they may also see value in drawing group boundaries that are inclusive of other ethnoracial minority groups, which experience related forms of discrimination from common elite sources.

Further, as minorities have gravitated toward each other, Whites, who sit at the top of the American racial hierarchy (Bonilla-Silva, 2001; Feagin, 2013; Kim, 1999; Omi & Winant, 2014), have simultaneously juxtaposed themselves against all others not in the dominant racial stratum (Perez, 2020). Political scientists note the contemporary relevance of White identity in shaping White public opinion (Jardina, 2019, 2020; Sides et al., 2019) and White racial linked fate increasing political participation (Berry et al., 2019). In comparison with minorities who respond to changing levels of discrimination with more *solidaridad*, Marsh and Ramirez (2019) argue that Whites respond to their perceived loss of status with a sense of *linked anxiety*. The emergence of a White identity suggests that the group is reinforcing within-group boundaries to protect their dominant status in society from perceived threats (Abascal, 2020; Abrajano & Hajnal, 2015). While still acknowledging individual racial group experiences and uniqueness, there has been a non-mutually exclusive distinction between a dominant White group and a non-White, minority group inclusive of African Americans, Asian Americans, and Latina/os.

In addition to this White and non-White juxtaposition, even with the changing demographic composition of the U.S., scholars have cautioned about the limits of demographic change (Fraga, 2018; Wong, 2018). Despite strength in increasing numbers, Schmidt et al. (2009) note that minorities have yet to reach parity with Whites in terms of participation, representation, influence, and realization of policies which advance racial equality. Even as an increasing share of the U.S. population, minorities are still likely to continue living in a political system characterized by White dominance. Following this, we argue it is especially important to look at how inter-group solidarity via feelings of minority linked fate, shape political behavior. We build on foundational work assessing how inter-group considerations influence public opinion (Bejarano et al., 2020; Gershon et al., 2019; Merseth, 2018; Perez, 2021b).

## Inter-Racial Minority Linked Fate as a Basis for Political Behavior

We now turn to theorize about how minority linked fate shapes the political participation of African Americans, Latina/os, and Asian Americans. We place inter-racial linked fate alongside intra-racial linked fate as people of color are not solely guided by their intra-racial group solidarity (Perez, 2021a, 2021b). As complementary expressions, we ground our mobilizing expectations of minority linked fate within social identity theory. Accordingly, we contend the complementary insight gained from examining inter-racial linked fate can be observed in the domain of political behavior.

Social identity theory describes the innate human tendency to classify individuals primarily on the basis of who belongs to your group (Tajfel & Turner, 1979). A natural outgrowth of this cognitive categorization is for members to develop in-group bias while distancing from out-groups (Turner et al., 1987). Individuals can further hold multiple in-group identities at the subgroup and superordinate levels (Gaertner et al., 2000); this suggests that one can hold both a racial group identity and a broader minority identity. The current political context further provides a possibility for minority individuals to shift their perspective to one where their minority identity, under which their within-group racial identities are nested, are both prominent (Perez, 2021a). These identities are complementary rather than zero-sum.

This minority superordinate identity has political significance. Applying social identity theory, individuals who derive a positive self-image from their in-group are motivated to defend and strengthen the social image of their group (Tajfel & Turner, 1979). This theoretical mechanism of acting on behalf of the in-group occurs at both the subgroup and superordinate levels (Gaertner et al., 2000). To the degree that intra-racial and inter-racial linked fate capture this psychological mechanism, individuals valuing and adhering to both of these group-based resources should be similarly mobilized. Thus, feelings of attachment toward minorities such as Asian Americans, African Americans, or Latina/os motivate the marginalized to take political action on behalf of this overarching minority group. Those who perceive minority linked fate take courses of preventative or responsive political action in order to restore or uplift the image of all minorities (Perez, 2015; Sanchez, 2006; Tajfel & Turner, 1979). Perceiving relative disadvantage to Whites, there is sufficient motivation for Asian Americans, African Americans, and Latina/os to seek change through political participation in order to defend, protect, and boost the status and collective goals of minorities as a whole (Branscombe et al., 1999; Doosje et al., 2002; Leach et al., 2010; Mason, 2018; Perez, 2021b). Political activity becomes particularly likely among those with the strongest sense of minority linked fate because their sense of self-worth and esteem derives from the social standing of this superordinate minority group (Tajfel & Turner, 1979).

We argue that Latina/o, Asian, and African Americans who perceive minority linked fate, a sense of solidarity which spans across individual racial groups, have the strongest affective investment in the image of minorities as a whole. Those who feel particularly tied to the fate of other minorities, in turn, are motivated to take political action on behalf of this minority collective. Latina/o, African American, and Asian Americans see their political participation as advancing minorities’ social status and goals. That is, they themselves do better if minorities as a whole do better as well. We thus hypothesize that:

### H1

African Americans, Latina/os, and Asian Americans who hold stronger feelings of minority (inter-racial) linked fate are more likely to participate in politics across a wide range of actions including conventional and unconventional political activities.

## Variation in Minority Linked Fate’s Influence on Latina/o, Asian, and African American Political Participation

We further theorize about the extent to which minority linked fate mobilizes African Americans, Latina/os, and Asian Americans. We expect the influence of inter-racial linked fate, although still positive, to be weaker among African Americans due to the contributions of their intra-racial linked fate. Inter-racial linked fate, as with traditional notions of intra-racial linked fate, functions differently across groups as a reflection of a racially stratified society, which imposes differential constraints on individuals (Masuoka & Junn, 2013). Among African Americans, intra-racial linked fate is unique because of the group’s history of enslavement, segregation, discrimination, and deprivation (Dawson, 1994). Under the social identity framework, the uniqueness of the Black experience centers the use and presence of intra-racial linked fate as a form of affirming positive distinctiveness (Tajfel & Turner, 1979). Their historical circumstances have produced a commitment to group solidarity and developed “racialized social pressure” to adhere to norms about appropriate group behavior (White et al., 2014). The enforcement of African American group norms arises out of the experience with de jure and de facto segregation that does not have a parallel among Asian Americans and Latina/os (White & Laird, 2020).^3^ Moreover, Latina/os’ and Asian Americans’ relatively more positive social and economic experiences compared to African Americans weakens their reliance on intra-racial, group-based considerations (Chong & Kim, 2006; Sears & Savalei, 2006) and is therefore less central to the political behavior of Latina/os and Asian Americans relative to African Americans.

To the degree that African Americans are influenced by a within-group history of discrimination and strongly held within-group social norms, the mobilizing influence of a minority linked fate for African Americans may be more subdued. We thus expect any additional influence of inter-racial linked fate beyond that of intra-racial linked fate to be more pronounced among Latina/os and Asian Americans compared to that of African Americans. Our second hypothesis states:

### H2

Minority (inter-racial) linked fate has a stronger positive relationship to political participation among Latina/o and Asian Americans relative to African Americans.

## Data: The 2016 Collaborative Multiracial Post-Election Survey

To test our theoretical expectations, we leverage the 2016 Collaborative Multiracial Post-Election Survey (CMPS, Barreto et al., 2017). In 2016, the CMPS collected large, national samples of Asians (n=3006), African Americans (n=3102), and Latina/os (n=3003). This allows for researchers to comparatively examine the political behavior of racial minorities. The survey was also conducted in five languages allowing individuals to select the language they were most comfortable with.

This included Chinese (simplified), Chinese (traditional), Korean, Vietnamese, Spanish, and English.

Survey methodology is fully laid out in Barreto et al. (2018). All interviews were self- administered online between December 3, 2016 and February 15, 2017. Data samples included large numbers of registered and non-registered voters. The survey had an effective response rate of 9.9%. We join the growing scholarship which has looked at the political participation of racial minorities separately and comparatively using the 2016 CMPS (for example see: Chan & Phoenix, 2020; Greene et al., 2020; Gutierrez et al., 2019; Masuoka et al., 2019).

## Variables of Interest

### Independent Variable

Our main explanatory variable is minority linked fate. It is defined as “the idea that ethnoracial minorities might share a sense of commonality that extends beyond their particular ethnoracial group to other ethnoracial groups” (Gershon et al., 2019). We utilize their same measure for minority or inter-racial linked fate: “What happens to racial and ethnic minorities in this country will have something to do with what happens in your life” (0 = not at all; 0.333 = not much; 0.667 = some; 1 = a lot). This is conceptually different from intra-racial linked fate where one’s own life chances are linked to that of others within their racial group (Dawson, 1994).

### Dependent Variable

Our outcome variable is political participation. We posit that minority linked fate has positive associations with political action broadly but still see it best fit to assess political participation by different modes of activities. We analyze a self-reported measure of voter turnout in the 2016 general election. The question asks: “This year a lot of people said they did NOT vote in the election, because they were just too busy, not that interested in politics, or frankly don’t like their choices. How about you? Would the official vote records for [STATE] indicate that you voted in (the) 2016 election, or like many people, did you skip this one?” (1 = Yes, I voted; 0 = No, I did NOT vote). We also measure participation in five conventional (electoral) activities. This includes whether respondents over the span of a year worked for or donated to a candidate, campaign, or party organization; volunteered on a campaign; contacted an elected representative or government official; worked or cooperated with others to try to solve a problem affecting your city or neighborhood; or attended a meeting to discuss issues facing the community. We also look at three unconventional (non-electoral) activities such as having protested; boycotted a company or product for political reasons; or signed a petition regarding an issue or problem that concerned them.

### Control Variables

We account for standard socioeconomic demographics such as education, income, age, gender, and involvement in civic organizations as a proxy for the possible development of civic skills (Verba et al., 1995). We also control for respondents’ place of birth, as well as the composition of co-ethnics living in respondents’ neighborhoods. This is because previous work has found that community context shapes linked fate and political behavior (Bledsoe et al., 1995; Cohen & Dawson, 1993; Gay, 2004). We also control for variation in political orientations such as partisanship, strength of partisan identity, perceptions of political efficacy, and whether interviewees had been recruited to participate in politics. Most importantly, we include a traditionally used measure of intra-racial linked fate in our models. The standard wording of intra- racial (within-group) linked fate question is as follows: “Do you think what happens generally to people in your own racial/ethnic group in this country will have something to do with what happens in your life?” (1 = yes & it will affect me a lot; 0.667 = yes & it will affect me some; 0.333 = yes & it will affect me not very much; 0 = no).

## Methods

We begin with descriptive statistics looking at the mean levels of inter-racial and intra-racial linked fate within and across African Americans, Asian Americans, and Latina/os. We then look at the association between minority linked fate and (1) Voting, (2) Conventional Participation, and (3) Unconventional Participation across the three groups of interest. We run Poisson models for our conventional and unconventional political actions since these dependent variables have count distributions; voting models with binary outcomes are run with logistic regressions. For brevity, full regression output is not all reported but is transparent in the various appendices. In most cases, only the change in predicted probabilities based on regression coefficients are displayed.

## Descriptive Statistics

Table 1 displays the mean level of minority (inter-racial) and within-group (intra-racial) linked fate across our three racial groups of interest. African Americans, Latina/os, and Asian Americans all have a stronger sense of minority linked fate than within-group linked fate (all p < 0.01). African Americans report higher levels of both inter-racial and intra-racial linked fate compared to Asian Americans and Latina/os (all p < 0.01)^4^^,^ 5^,^ 6^,^ 7

**Table 1:** Comparing feelings of inter-racial and intra-racial linked fate among minority groups

	n	Inter-racial linked fate	Intra-racial linked fate
African Americans	3102	0.69	0.51
Latina/o Americans	3003	0.53	0.42
Asian Americans	3006	0.58	0.43
Minorities (All)	9111	0.6	0.46

Inter-racial and Intra-racial Linked Fate are both coded between 0 (Lowest)-1 (Highest)

## Results

**Fig. 1:**
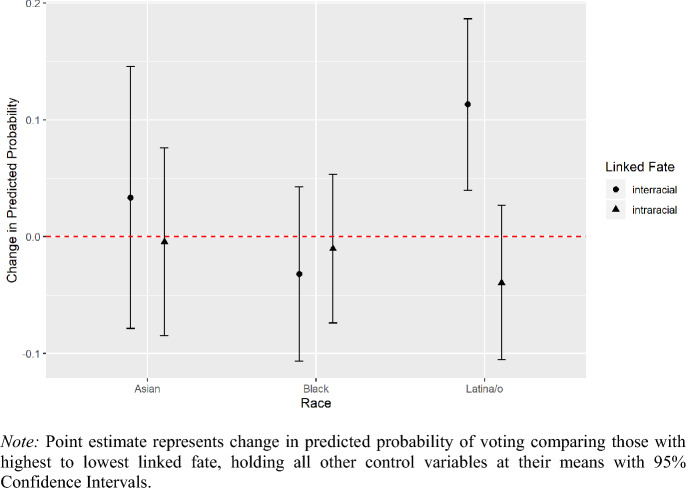
Inter-racial and intra-racial linked fate on voter turnout in 2016

We begin by looking at the relationship between minority linked fate and turning out to vote in the 2016 Presidential Election. Full regression model results are reported in the appendix. We present the change in predicted probabilities instead in Fig. 1. The results note that both forms of linked fate are neither substantially nor significantly associated with Asian or African American turnout in 2016, which comport to results from Chong and Rogers (2005), Laniyonu (2019), and Wong et al. (2005) that racial group-based indicators are not related to ballot-box behavior. While traditional notions of linked fate do not positively associate with Latina/o 2016 turnout, Latina/os who perceive the strongest sense of minority linked fate are about 11 percentage points more likely than those who do not feel any sense of inter-racial linked fate to have turned out to vote in 2016.

**Fig. 2:**
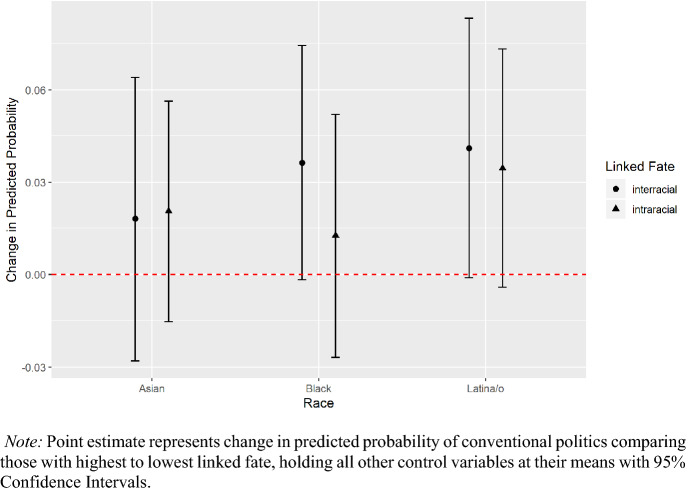
Intra-racial and inter-racial linked fate on conventional political activity

Next, we analyze the relationship between different types of linked fate and conventional political participation, apart from activities that are done exclusively at the ballot box. Figure 2 presents the change in predicted probabilities comparing individuals with the highest versus the lowest perceptions of within and across-group linked fate separately, holding all controls at their means. Full regression models again are reported in the appendix. The results note that both minority and intra-racial linked fate have a positive relationship to conventional political participation for Latina/os. However, as was the case for voting, inter-racial linked fate has a slightly stronger association (~ + 4 points, p = 0.07) with this mode of Latina/o political involvement than intra-group linked fate (~ + 3 points). Asian Americans are neither shaped by minority nor traditional notions of linked fate to take conventional political actions. Among African Americans, we find that minority linked fate has a positive relationship to their conventional political participation and one that is stronger than that of intra-racial linked fate. However, since the p-value for the minority linked fate coefficient is p = 0.14 and is above the alpha of 0.1 level, this is not a result we spotlight.

**Fig. 3:**
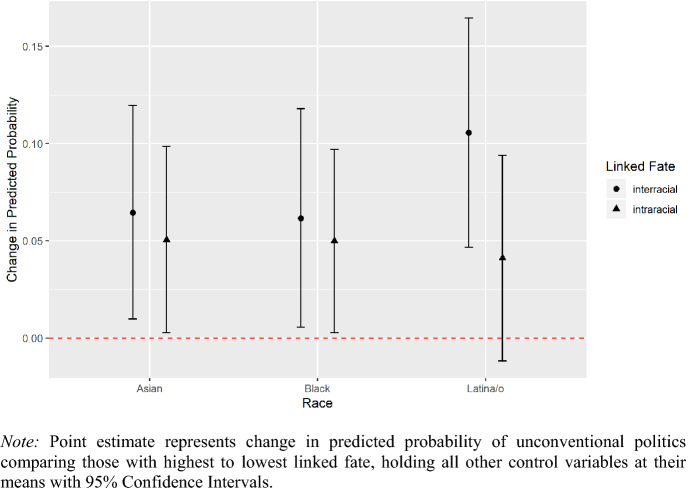
Inter-racial and intra-racial linked fate on unconventional political involvement

The key findings of this paper demonstrate that minority linked fate is most strongly and significantly related to unconventional forms of political participation such as political consumerism, petition signing, and attending protests or rallies, for Latina/os, Asian Americans, and African Americans. The results displayed in Fig. 3 show the change in predicted probability of unconventional political action comparing individuals with the highest by the lowest degrees of two different types of linked fate, inter-racial (minority) and intra-racial (within-group). In terms of unconventional political action, both types of linked fate are associated with more participation among African Americans. Inter-racial linked fate is slightly more positively related to contentious action (~+6 points) than intra-racial linked fate (~+5 points). For Asian Americans, minority linked fate is also a bit more correlated with unconventional political activity (~+6.5 points) than intra-racial linked fate (+~5 points). Figure 3 also notes that the estimated relationship between minority linked fate and unconventional participation more than doubles that of within-group linked fate among Latina/os. Thinking more strongly in terms of an inter-racial linked fate increases Latina/os probability of participating in unconventional activities by about 10.5 percentage points. Latina/os who think in terms of linked fate within their own racial group are still more likely to take contentious activity such as protests, product boycotts, or petitions, but only by about 4 points.

For robustness, we looked at the relationship between minority linked fate and unconventional political participation for Asian Americans, Latina/os, African Americans across different model specifications. The full results are in the supplemental appendix. For all groups, again, minority linked fate and intra-racial linked fate are positively associated with contentious political action. Even though we posited in our theory that intra-racial linked fate and inter-racial linked fate are complementary heuristics, it does seem to be the case that the latter has a slightly stronger relationship to this specific type of political participation than the influence of the former.

Minority linked fate may be more strongly associated with unconventional political activity because relative to traditional notions of intra-racial linked fate, inter-racial minority linked fate signals participation on behalf of a more expansive and superordinate identity group. The motivation of those who express inter-racial linked fate is not just in reference to those within their own individual racial in-group but additionally to those across other racial minority groups outside of their own. As such, those with a greater sense of inter-racial minority linked fate identify more opportunities to, for example—attend protests that center the needs of minorities more broadly inside and outside of their own racial in-group. Further, in line with our theoretical expectations, the impact of minority linked fate among Latina/os and Asian Americans somewhat exceeds that of the relationship between minority linked fate and unconventional political action for African Americans.

## Discussion

Using a unique data set which oversamples for minorities, the results from the 2016 Collaborative Multiracial Post-Election Survey provide support for our theoretical expectations denoted in our first hypothesis. However, this is conditional on the type of political participation under scrutiny. We find that minority linked fate is a unique mobilizer to unconventional and direct, system-challenging forms of political action across Latina/os, Asian Americans, and African Americans, and to a lesser degree for electoral participation. Providing support for our second hypothesis, minority linked fate for Latina/o and Asian American respondents is slightly more strongly related to unconventional action than it is for African Americans. We urge future research to further interrogate the link between group solidarity and turnout for Latina/os as our findings suggest minority linked fate rather than intra-racial linked fate was positively related to their voter behavior, at least in the context of 2016. To the degree that a minority identity and related sense of linked fate is taking root in the contemporary political context, it may be of value to further explore if and how each racial group develops and internalizes this politically nascent identity.^8^

Across all three groups, minority linked fate has a positive and significant relationship with unconventional participation. The orientation and preference minority groups have toward unconventional activity is consistent with prior patterns that racial group solidarity relates particularly to more contentious, direct modes of political activity (Chong & Rogers, 2005). Moreover, Anoll (2018) suggests that groups with histories of disenfranchisement and segregation develop social norms placing greater value on unconventional political participation. It is important to note that this relationship toward system-challenging action exists not just for subgroup but superordinate groups as well. As such, our theoretical and empirical contributions suggest the importance of minority linked fate in advance of the political future (see also: Perez, 2021a)—one that follows recent patterns of unconventional political activity among those of different individual racial group identities but with a complementary, superordinate identity dimension. With the ever-present threats to the franchise, jump started with the dismissal of voting protections in Shelby County v. Holder (2013) and attacks to voting rights following the 2020 election, electoral means of pressuring for government responsiveness may be prioritized less, leaving minorities to pursue unconventional methods as their dominant recourse.

## Conclusion

Overall, these findings make a novel contribution to our understanding of political behavior among Latina/os, Asian Americans, and African Americans. We suggest that they may not be solely motivated by intra-group considerations, but also by their shared non-dominant social status as minorities. Empirically, while we have made use of a reliable and large oversample of racial minorities to test our theory, we are not able to make causal claims. We insist that an avenue for future research will be to prime our independent variable of interest in experimental settings, following the lead of Perez (2021b) who does so for a “people of color” socio-political identity. For now, we make only associational claims on the relationship between minority linked fate and political behavior.

We advance developments in seminal research on group-based resources and indicators of political behavior (Chong & Rogers, 2005; Dawson, 1994; Sanchez, 2006; Wong et al., 2005). Here, we illuminate not only the importance of intra-racial (within-group) linked fate but how minority (inter-racial) linked fate can also influence political action, especially protest behavior. Protest mobilization on the basis of minority linked fate is especially crucial because recent research shows that even though protest activity is costly, legislators can be receptive to the preferences of protestors and particularly to that of racial minorities (Gause, 2020). We add to the scholarly understanding of minority linked fate initiated by Bejarano et al. (2020); Gershon et al. (2019); and Merseth (2018) as we think about this group-based heuristic’s continued importance in incentivizing the marginalized to stand together with other minorities. Expanding on social identity theory, we theorize about the animating force behind minority linked fate. While subgroup identities center the immediate racial group itself, we suggest a minority identification reflects a real-world social cleavage between Whites and non-Whites that serves as an additional cue in helping individuals make sense of their politics (Conover, 1988). We thus hypothesized that those who feel particularly tied to the fate of other minorities take political action on behalf of a minority collective as a form of advancing the status and political interests of the broader minority community. Aligned with Perez (2021a), our findings highlight the complexity in how minority individuals use multiple group-based resources to navigate the political world.

This study’s findings and contribution come as a reflection of the contemporary period in which people of color continue to be made aware of their precarious position and future in American politics. A sense of minority linked fate thus helps us understand why different racial groups come together to take contentious political action and make demands on government. The recent, nationwide Black Lives Matter protests in 2020 have responded to the murder of African Americans at the hands of law enforcement and reflect the Black community’s enduring calls for decent human treatment and legislative change. Despite a clear disproportion in who is negatively affected by police brutality, significant numbers of Latina/os and Asian Americans voice their support and commitment to the goals of Black Lives Matter in the form of a multiracial coalition centered around bringing justice for George Floyd, Breonna Taylor, and many others. In these dire moments that scrutinize our nation’s commitment to its democratic values, we may wonder why non-Black minorities take to the streets alongside African Americans to pressure the government to enact substantive institutional change. That a sense of minority inter-racial linked fate mobilizes individuals of marginalized groups to make demands on the system through specific forms of participation, including protest, informs our understanding of the complex group-based incentives that mobilize racial minorities into politics in this current political moment. A sense of minority linked fate should remain, if not strengthen, into the future provided that political environments and conditions signal minorities of their subordinate status to Whites.^9^

### Electronic supplementary material

Below is the link to the electronic supplementary material.

Supplementary file1 (DOCX 28 kb)

## Data Availability

The CMPS 2016 is currently embargoed and eventually will be made public on ICPSR.

## Appendix

See Tables 2, 3, 4.

**Table 2:** Inter and intra-racial linked fate on 2016 turnout

*Dependent variable: 2016 voter turnout*			
	African American	Latina/o	Asian American
Minority linked fate (inter)	− 0.161	0.630***	0.149
	(0.201)	(0.219)	(0.250)
Linked fate (intra)	− 0.054	− 0.216	− 0.017
	(0.158)	(0.187)	(0.191)
Age	5.971***	5.900***	5.324***
	(0.423)	(0.535)	(0.464)
Income	1.816***	1.548***	1.149***
	(0.244)	(0.246)	(0.214)
Education	2.572***	3.542***	2.046***
	(0.310)	(0.339)	(0.323)
Female	0.397***	− 0.085	− 0.289**
	(0.123)	(0.134)	(0.127)
Not born in the U.S.	− 0.352	0.394**	0.046
	(0.315)	(0.170)	(0.135)
Strength of partisanship	0.521***	0.407***	0.429***
	(0.051)	(0.057)	(0.063)
Conservative	− 0.173	− 0.112	0.006
	(0.152)	(0.163)	(0.165)
Republican	− 1.126***	− 0.524***	− 0.230
	(0.272)	(0.178)	(0.178)
Interest in politics	2.113***	1.961***	1.746***
	(0.212)	(0.230)	(0.246)
Internal efficacy	− 0.057	− 0.252	0.417*
	(0.189)	(0.227)	(0.244)
External efficacy	0.035	0.899***	0.163
	(0.223)	(0.245)	(0.258)
Recruitment	0.097	0.031	0.340**
	(0.124)	(0.143)	(0.153)
Co-ethnic neighborhood	0.461**	− 0.066	− 0.378
	(0.183)	(0.206)	(0.256)
Civic org involvement	− 0.037	0.261	0.369
	(0.204)	(0.239)	(0.244)
Constant	− 6.405***	− 6.242***	− 5.674***
	(0.346)	(0.391)	(0.396)
Observations	2444	2105	1774
Log likelihood	− 1024.488	− 882.335	− 838.432
Akaike inf. crit	2082.976	1798.671	1710.863

Logistic regression coefficients with standard errors in parentheses

*p = 0.1; **p < 0.05; ***p = 0.01

**Table 3:** Inter and intra-racial linked fate on conventional political participation

*Dependent variable: conventional political participation*			
	African American	Latina/o	Asian American
Minority linked fate (inter)	0.275	0.345*	0.189
	(0.188)	(0.201)	(0.231)
Linked fate (intra)	0.094	0.283*	0.197
	(0.146)	(0.166)	(0.182)
Age	0.054	0.190	0.113
	(0.325)	(0.396)	(0.391)
Income	0.336*	0.244	0.060
	(0.179)	(0.197)	(0.211)
Education	0.139	0.240	− 0.106
	(0.258)	(0.276)	(0.316)
Female	− 0.129	− 0.115	− 0.164
	(0.100)	(0.109)	(0.118)
Not born in the U.S.	− 0.138	− 0.089	− 0.081
	(0.228)	(0.123)	(0.118)
Strength of partisanship	− 0.015	0.047	0.003
	(0.045)	(0.052)	(0.058)
Conservative	− 0.080	0.049	− 0.018
	(0.143)	(0.151)	(0.164)
Republican	0.118	− 0.049	− 0.005
	(0.216)	(0.159)	(0.166)
Interest in politics	1.005***	1.094***	1.688***
	(0.210)	(0.237)	(0.269)
Internal efficacy	0.025	0.149	0.091
	(0.156)	(0.179)	(0.214)
External efficacy	− 0.060	− 0.023	0.103
	(0.188)	(0.205)	(0.227)
Recruitment	0.305***	0.328***	0.318**
	(0.098)	(0.110)	(0.125)
Co-ethnic neighborhood	0.151	− 0.130	0.050
	(0.155)	(0.189)	(0.250)
Civic org involvement	1.232***	1.239***	1.340***
	(0.126)	(0.140)	(0.151)
Constant	− 3.258***	− 3.694***	− 3.699***
	(0.277)	(0.311)	(0.354)
Observations	2527	2388	2266
Log likelihood	− Inf.000	− Inf.000	− Inf.000
Akaike inf. crit	Inf.000	Inf.000	Inf.000

Poisson regression coefficients with standard errors in parentheses

*p = 0.1; **p < 0.05; ***p = 0.01

**Table 4:** Inter and intra-racial linked fate on unconventional political participation

*Dependent variable: unconventional political participation*			
	African American	Latina/o	Asian American
Minority linked fate (inter)	0.335**	0.548***	0.417**
	(0.164)	(0.163)	(0.195)
Linked fate (intra)	0.251**	0.212	0.312**
	(0.127)	(0.133)	(0.151)
Age	− 0.713**	− 0.350	− 0.366
	(0.288)	(0.331)	(0.339)
Income	0.337**	0.214	0.117
	(0.156)	(0.159)	(0.174)
Education	0.412*	0.677***	0.077
	(0.223)	(0.224)	(0.263)
Female	0.044	0.081	0.097
	(0.090)	(0.090)	(0.100)
Not born in the U.S.	− 0.318	− 0.262**	− 0.171*
	(0.209)	(0.103)	(0.099)
Strength of partisanship	0.690***	− 0.181	− 0.130
	(0.116)	(0.152)	(0.217)
Conservative	− 0.034	0.476***	0.785***
	(0.136)	(0.124)	(0.134)
Republican	0.047	0.054	0.115**
	(0.040)	(0.041)	(0.048)
Interest in politics	− 0.248*	− 0.130	− 0.017
	(0.134)	(0.133)	(0.144)
Internal efficacy	− 0.079	− 0.200	− 0.325**
	(0.213)	(0.141)	(0.152)
External efficacy	0.896***	1.152***	1.232***
	(0.177)	(0.189)	(0.215)
Recruitment	0.142	0.273*	0.310*
	(0.135)	(0.147)	(0.178)
Co-ethnic neighborhood	− 0.380**	− 0.282*	− 0.347*
	(0.166)	(0.167)	(0.195)
Civic org involvement	0.316***	0.312***	0.301***
	(0.085)	(0.089)	(0.106)
Constant	− 2.797***	− 3.237***	− 3.150***
	(0.241)	(0.253)	(0.295)
Observations	− Inf.000	2388	2266
Log likelihood	Inf.000	− Inf.000	− Inf.000
Akaike inf. crit	0.335**	Inf.000	Inf.000

Poisson regression coefficients with standard errors in parentheses

*p = 0.1; **p < 0.05; ***p = 0.01

## Footnotes

1 We use “across-group” interchangeably with “inter-group” to refer to minority linked fate. We use “within-group” synonymously with “intra-group” to refer to traditional notions of linked fate.

2 We note that scholars have cautioned about extrapolating group-based linked fate frameworks, which should be treated as a “hypothesis rather than an assumption” for non-Black minorities (Junn & Masuoka, 2008; See also: Dawson, 1994; Lee, 2008; McClain et al., 2009; Sears & Savalei, 2006).

3 This is not to say that segregation and discrimination have not impacted the social identity of non-Black minorities, we merely posit that identification, solidarity, and group norms arising from the groups’ histories are more crystalized for African Americans.

4 The positive difference between African Americans’ inter-racial and intra-racial linked fate compared to Latina/os and Asian Americans are significant at the p < 0.01 level. While there is also a significant difference between Asian Americans’ and Latina/os’ level of minority linked fate, their sense of within-group linked fate are not distinguishable from 0.

5 We also looked at the correlation between inter-racial and intra-racial linked fate (Latina/os, r = 0.56; Asian Americans, r = 0.45; African Americans, r = 0.43). While high, we still maintain that minority linked fate and standard notions of within-group linked fate are conceptually distinct. Even so, in terms of operationalization and measurements, previous studies have used both variables together in one model specification assessing minorities’ political attitudes (Merseth, 2018).

6 Future work might assess how context shapes perceptions of minority linked fate. We find preliminarily that African American, Latina/o, and Asian Americans who live in neighborhoods with a larger proportion of co-ethnics are more likely to express minority linked fate.

7 We, additionally, examined if Latina/o and Asian American national origin groups hold varying levels of minority linked fate as some segments of Latina/o and Asian American pan-ethnic groups have different experiences of racialization. Cubans have statistically significantly lower levels of minority linked fate (0.43) relative to Puerto Ricans (0.52) and Latina/os as a whole (0.54). No Asian national origin group significantly deviates from their Asian pan-ethnic group (e.g., Asian American = 0.58; Chinese = 0.58; Japanese = 0.56).

8 We also recognize the limitations of this finding (that minority linked fate is positively related to turnout among Latina/os) given the uniqueness of the 2016 election and since the results rely only on one cross-sectional survey.

9 Replication materials are available here: https://dataverse.harvard.edu/dataset.xhtml?persistentId=doi%3A10.7910%2FDVN%2FTAZPWZ&version=DRAFT.

## References

[CR1] Abascal M (2020). Contraction as a response to group threat: Demographic decline and whites’ classification of people who are ambiguously white. American Sociological Review.

[CR2] Abrajano M, Hajnal ZL (2015). White backlash: Immigration, race, and American politics.

[CR3] Aldrich, J. H. (1993). Rational choice and turnout. *American Journal of Political Science*, 246–278.

[CR97] Anoll AP (2018). What makes a good neighbor? Race, place, and norms of political participation. American Political Science Review.

[CR5] Barreto, M., Frasure-Yokley, L., Vargas, E. D., & Wong, J. (2017). *The Collaborative Multiracial Post-Election Survey (CMPS), 2016*.

[CR6] Barreto MA, Frasure-Yokley L, Vargas ED, Wong J (2018). Best practices in collecting online data with Asian, Black, Latino, and White respondents: Evidence from the 2016 Collaborative Multiracial Post-election Survey. Politics, Groups, and Identities.

[CR7] Barvosa E (2008). Wealth of selves: Multiple identities, mestiza consciousness, and the subject of politics.

[CR8] Bejarano C, Brown NE, Gershon SA, Montoya C (2020). Shared identities: Intersectionality, linked fate, and perceptions of political candidates. Political Research Quarterly.

[CR9] Beltrán C (2010). The trouble with unity: Latino politics and the creation of identity.

[CR10] Berry, J.A., Ebner, D., & Cornelius, M. (2019). White identity politics: linked fate and political participation. *Politics, Groups, and Identities*, *9*(3).

[CR11] Bledsoe, T., Welch, S., Sigelman, L., & Combs, M. (1995). Residential context and racial solidarity among African Americans. *American Journal of Political Science*, 434–458.

[CR12] Bonilla-Silva E (2001). White supremacy and racism in the post-civil rights era.

[CR13] Bowler S, Segura G (2011). The future is ours: Minority politics, political behavior, and the multiracial era of American politics.

[CR14] Branscombe NR, Schmitt MT, Harvey RD (1999). Perceiving pervasive discrimination among African Americans: Implications for group identification and well-being. Journal of Personality and Social Psychology.

[CR15] Campbell A, Converse PE, Miller WE, Stokes DE (1960). The American voter.

[CR16] Chan, N. K, Kim, J. Y., & Leung, V. (2021). COVID-19 and Asian Americans: How elite messaging and social exclusion shape partisan attitudes. *SocArXiv*. 10.31235/osf.io/dvm7r.

[CR17] Chan, N. K., & Phoenix, D. L. (2020). The ties that bind: Assessing the effects of political and racial church homogeneity on Asian American Political Participation. *Politics and Religion*, 1–32.

[CR18] Chong D, Kim D (2006). The experiences and effects of economic status among racial and ethnic minorities. American Political Science Review.

[CR19] Chong D, Rogers R (2005). Racial solidarity and political participation. Political Behavior.

[CR20] Cohen, C. J., & Dawson, M. C. (1993). Neighborhood poverty and African American politics. *American Political Science Review*, 286–302.

[CR21] Conover, P. J. (1988). The role of social groups in political thinking. *British Journal of Political Science*, 51–76.

[CR22] Craig MA, Richeson JA (2012). Coalition or derogation? How perceived discrimination influences intraminority intergroup relations. Journal of Personality and Social Psychology.

[CR23] Dawson MC (1994). Behind the mule: Race and class in African American politics.

[CR24] Doosje B, Spears R, Ellemers N (2002). Social identity as both cause and effect: The development of group identification in response to anticipated and actual changes in the intergroup status hierarchy. British Journal of Social Psychology.

[CR25] Dowe, P. K. F., Franklin, S. M., & Carter, N. M. (2018). Policy symmetry and cross-racial linked fate in the early years of the Obama presidency. *Politics, Groups, and Identities*, 1–27.

[CR26] Feagin J (2013). Systemic racism: A theory of oppression.

[CR27] Fraga BL (2018). The turnout gap: Race, ethnicity, and political inequality in a diversifying America.

[CR28] Gaertner SL, Dovidio JF, Anastasio PA, Bachman BA, Rust MC (1993). The common ingroup identity model: Recategorization and the reduction of intergroup bias. European Review of Social Psychology.

[CR29] Gaertner SL, Dovidio JF, Samuel G (2000). Reducing intergroup bias: The common ingroup identity model.

[CR30] Garcia, S. E. (2020). Where did BIPOC come from? *New York Times*. Retrieved from https://www.nytimes.com.

[CR31] Garcia-Rios S, Pedraza F, Wilcox-Archuleta B (2019). Direct and indirect xenophobic attacks: Unpacking portfolios of identity. Political Behavior.

[CR32] Gause, L. (2020). Revealing issue salience via costly protest: How legislative behavior following protest advantages low-resource groups. *British Journal of Political Science*. 1–21.

[CR33] Gay, C. (2004). Putting race in context: Identifying the environmental determinants of Black racial attitudes. *American Political Science Review*, 547–562.

[CR34] Gershon SA, Montoya C, Bejarano C, Brown N (2019). Intersectional linked fate and political representation. Politics, Groups, and Identities.

[CR35] Greene S, Gray G, Carter N, Block R (2020). Americanness and the “Other” Americans: An examination of the American identity and political behavior of racial and ethnic minorities in the United States. National Review of Black Politics.

[CR36] Griffin, R., Sides, J., & Tesler, M. (2019). Negative views of Asian people have risen in both parties. *Washington Post*. Retrieved from https://www.washingtonpost.com.

[CR37] Gutierrez A, Ocampo A, Barreto M, Segura G (2019). Somos Más: How racial threat and anger mobilized Latino voters in the Trump era. Political Research Quarterly.

[CR38] Jardina A (2019). White identity politics.

[CR39] Jardina, A. (2020). In-group love and out-group hate: White Racial attitudes in contemporary US elections. *Political Behavior*, 1–25.

[CR40] Jeung, R., Horse, A. Y., Popvic, T., & Lim, R. (2021). Stop AAPI Hate National Report. *Stop AAPI Hate*. Retrieved from https://secureservercdn.net/104.238.69.231/a1w.90d.myftpupload.com/wp-content/uploads/2021/03/210312-Stop-AAPI-Hate-National-Report-.pdf.

[CR41] Junn J, Masuoka N (2008). Asian American identity: Shared racial status and political context. Perspectives on Politics.

[CR42] Kaufmann KM (2003). Cracks in the rainbow: Group commonality as a basis for Latino and African-American political coalitions. Political Research Quarterly.

[CR43] Khalid, A. (2015). South Korea? Trump’s ‘Where are you from’ moment. *National Public Radio*. Retrieved from http://www.npr.org.

[CR44] Kim CJ (1999). The racial triangulation of Asian Americans. Politics & Society.

[CR45] Laniyonu A (2019). A comparative analysis of black racial group consciousness in the United States and Britain. Journal of Race, Ethnicity and Politics.

[CR46] Leach CW, Mosquera PMR, Vliek ML, Hirt E (2010). Group devaluation and group identification. Journal of Social Issues.

[CR47] Lee T (2008). Race, immigration, and the identity-to-politics link. Annual Review of Political Science.

[CR48] Leighley JE, Vedlitz A (1999). Race, ethnicity, and political participation: Competing models and contrasting explanations. The Journal of Politics.

[CR49] Lien PT (1994). Ethnicity and political participation: A comparison between Asian and Mexican Americans. Political Behavior.

[CR50] Lopez D, Espiritu Y (1990). Panethnicity in the United States: A theoretical framework. Ethnic and Racial Studies.

[CR51] Marsh WZ, Ramírez R (2019). Unlinking fate? Discrimination, group-consciousness, and political participation among Latinos and whites. Politics, Groups, and Identities.

[CR52] Mason L (2018). Uncivil agreement: How politics became our identity.

[CR53] Masuoka N (2006). Together they become one: Examining the predictors of panethnic group consciousness among Asian Americans and Latinos. Social Science Quarterly.

[CR54] Masuoka N (2008). Defining the group: Latino identity and political participation. American Politics Research.

[CR55] Masuoka N, Junn J (2013). The politics of belonging: Race, public opinion, and immigration.

[CR56] Masuoka N, Ramanathan K, Junn J (2019). New Asian American voters: Political incorporation and participation in 2016. Political Research Quarterly.

[CR57] McClain PD, Johnson Carew JD, Walton E, Watts CS (2009). Group membership, group identity, and group consciousness: Measures of racial identity in American politics?. Annual Review of Political Science.

[CR58] Merseth JL (2018). Race-ing solidarity: Asian Americans and support for Black Lives Matter. Politics, Groups, and Identities.

[CR59] Miller, A. H., Gurin, P., Gurin, G., & Malanchuk, O. (1981). Group consciousness and political participation. *American Journal of Political Science*, 494–511.

[CR60] Newman, B., Merolla, J. L., Shah, S., Lemi, D. C., Collingwood, L., & Ramakrishnan, S. K. (2019). The Trump effect: An experimental investigation of the emboldening effect of racially inflammatory elite communication. *British Journal of Political Science*, 1–22.

[CR61] Nguyen H (2017). Donald J. Trump and Asia: From campaign to government. Asian Affairs: an American Review.

[CR62] Nicholson HL, Carter JS, Restar A (2020). Strength in numbers: Perceptions of political commonality with African Americans among Asians and Asian Americans in the United States. Sociology of Race and Ethnicity.

[CR63] Omi M, Winant H (2014). Racial formation in the United States.

[CR64] Pan, J. C. (2020). A leftist future for Asian American politics. *New Republic*. Retrieved from http://www.newrepublic.com.

[CR65] Perez EO (2015). Xenophobic rhetoric and its political effects on immigrants and their co-ethnics. American Journal of Political Science.

[CR66] Perez, E. O. (2020). ‘People of color’ are protesting. Here’s what you need to know about this new identity. *Washington Post*. Retrieved from https://www.washingtonpost.com

[CR67] Pérez EO (2021). (Mis) Calculations, psychological mechanisms, and the future politics of people of color. Journal of Race, Ethnicity, and Politics.

[CR68] Perez EO (2021). Diversity’s child: People of color and the politics of identity.

[CR69] Reny, T. T., & Barreto, M. A. (2020). Xenophobia in the time of pandemic: Othering, anti-Asian attitudes, and COVID-19. *Politics, Groups, and Identities*, 1–24.

[CR70] Rosenstone SJ, Hansen J (1993). Mobilization, participation, and democracy in America.

[CR71] Rothschild, J. E. (2020). Identities, interest group coalitions, and intergroup relations. *Politics, Groups, and Identities*, 1–18.

[CR72] Samson FL (2014). Asian American Attitudes towards a US citizenship path for illegal immigrants: Immigration reform as racialised politics. Journal of Ethnic and Migration Studies.

[CR73] Sanchez GR (2006). The role of group consciousness in political participation among Latinos in the United States. American Politics Research.

[CR74] Sanchez GR (2008). Latino group consciousness and perceptions of commonality with African Americans. Social Science Quarterly.

[CR75] Sanchez GR, Masuoka N (2010). Brown-utility heuristic? The presence and contributing factors of Latino linked fate. Hispanic Journal of Behavioral Sciences.

[CR76] Sanchez G, Masuoka N, Abrams B (2019). Revisiting the brown-utility heuristic: A comparison of Latino linked fate in 2006 and 2016. Politics, Groups, and Identities.

[CR77] Schmidt R, Hero RE, Aoki AL, Alex-Assensoh YM (2009). Newcomers, outsiders, and insiders: Immigrants and American racial politics in the early twenty-first century.

[CR78] Sears DO, Savalei V (2006). The political color line in America: Many “peoples of color” or Black exceptionalism?. Political Psychology.

[CR80] Sexton J (2008). Amalgamation schemes: Antiblackness and the critique of multiracialism.

[CR81] Shaw TC, Foster KA, Combs BH (2019). Race and poverty matters: Black and Latino linked fate, neighborhood effects, and political participation. Politics, Groups, and Identities.

[CR82] Shingles RD (1981). Black consciousness and political participation: The missing link. American Political Science Review.

[CR83] Sides J, Tesler M, Vavreck L (2019). Identity crisis: The 2016 presidential campaign and the battle for the meaning of America.

[CR84] Tajfel H, Turner JC, Austin WG, Worchel S (1979). An integrative theory of inter-group conflict. The social psychology of inter-group relations.

[CR85] Tate K (1991). Black political participation in the 1984 and 1988 presidential elections. American Political Science Review.

[CR86] Turner JC, Hogg MA, Oakes PJ, Reicher SD, Wetherell MS (1987). Rediscovering the social group: A self-categorization perspective.

[CR87] Ufkes EG, Otten S, Van Der Zee KI, Giebels E, Dovidio JF (2012). Urban district identity as a common ingroup identity: The different role of ingroup prototypicality for minority and majority groups. European Journal of Social Psychology.

[CR88] Valdez Z (2011). Political participation among Latinos in the United States: The effect of group identity and consciousness. Social Science Quarterly.

[CR89] Verba S, Nie N (1972). Participation in America: Social equality and political participation.

[CR90] Verba S, Schlozman KL, Brady HE (1995). Voice and equality: Civic voluntarism in American politics.

[CR98] White, I. K., & Laird, C. N. (2020). *Steadfast Democrats: How social forces shape Black political behavior*. Princeton University Press.

[CR95] White IK, Laird CN, Allen TD (2014). Selling Out?: The politics of navigating conflicts between racial group interest and self-interest. American Political Science Review.

[CR91] Wong JS (2018). Immigrants, evangelicals, and politics in an era of demographic change.

[CR92] Wong JS, Lien PT, Conway MM (2005). Group-based resources and political participation among Asian Americans. American Politics Research.

[CR93] Wong JS, Ramakrishnan SK, Lee T, Junn J, Wong J (2011). Asian American political participation: Emerging constituents and their political identities.

[CR94] Zamora, S. (2011). Framing commonality in a multiracial, multiethnic Coalition. *Just Neighbors*, (pp. 299–322).

