# Supplementary material for: From Inter-Racial Solidarity to Action: Minority Linked Fate and African American, Latina/o, and Asian American Political Participation
Source: Polit Behav. 2021 Sep 25:1–23. Online ahead of print. doi: 10.1007/s11109-021-09750-6 (PMC8475384; doi:10.1007/s11109-021-09750-6)
Supplement: Supplementary file 1 — Supplementary file1 (DOCX 28 kb) [file 11109_2021_9750_MOESM1_ESM.docx]

# Supplemental Appendix Online

**Table D: Inter and Intra-Racial Linked Fate on African American Unconventional Political Participation**

| *Dependent variable: Unconventional Political Participation* | | | |
| --- | --- | --- | --- |
| African Americans Only | | | |
| Minority Linked Fate (Inter) | 0.468^***^ | 0.332^**^ | 0.335^**^ |
|  | (0.164) | (0.164) | (0.164) |
| Linked Fate (Intra) | 0.346^***^ | 0.278^**^ | 0.251^**^ |
|  | (0.126) | (0.127) | (0.127) |
| Age | -0.327 | -0.637^**^ | -0.713^**^ |
|  | (0.279) | (0.287) | (0.288) |
| Income | 0.433^***^ | 0.337^**^ | 0.337^**^ |
|  | (0.155) | (0.157) | (0.156) |
| Education | 0.483^**^ | 0.408^*^ | 0.412^*^ |
|  | (0.221) | (0.223) | (0.223) |
| Female | 0.015 | 0.051 | 0.044 |
|  | (0.089) | (0.090) | (0.090) |
| Not Born in the U.S. | -0.372^*^ | -0.325 | -0.318 |
|  | (0.208) | (0.209) | (0.209) |
| Civic Org Involvement | 0.919^***^ | 0.754^***^ | 0.690^***^ |
|  | (0.110) | (0.115) | (0.116) |
| Co-ethnic Neighborhood | 0.033 | -0.022 | -0.034 |
|  | (0.134) | (0.136) | (0.136) |
| Strength of Partisanship |  | 0.047 | 0.047 |
|  |  | (0.040) | (0.040) |
| Conservative |  | -0.247^*^ | -0.248^*^ |
|  |  | (0.134) | (0.134) |

| Republican |  | -0.101 | -0.079 |
| --- | --- | --- | --- |
|  |  | (0.213) | (0.213) |
| Interest in Politics |  | 0.938^***^ | 0.896^***^ |
|  |  | (0.177) | (0.177) |
| Internal Efficacy |  | 0.135 | 0.142 |
|  |  | (0.136) | (0.135) |
| External Efficacy |  | -0.375^**^ | -0.380^**^ |
|  |  | (0.166) | (0.166) |
| Recruitment |  |  | 0.316^***^ |
|  |  |  | (0.085) |
| Constant | -2.504^***^ | -2.773^***^ | -2.797^***^ |
|  | (0.212) | (0.240) | (0.241) |
| Observations | 2,527 | 2,527 | 2,527 |
| Log Likelihood | -Inf.000 | -Inf.000 | -Inf.000 |
| Akaike Inf. Crit. | Inf.000 | Inf.000 | Inf.000 |

*Note*: Poisson regression coefficients with standard errors in parentheses. *p=0.1; **p<0.05;

***p=0.01.

# Table E: Inter and Intra-Racial Linked Fate on Latina/o Unconventional Political Participation

| *Dependent variable: Unconventional Political Participation* | | | |
| --- | --- | --- | --- |
|  |  | Latina/os Only |  |
| Minority Linked Fate (Inter) | 0.772^***^ | 0.572^***^ | 0.548^***^ |
|  | (0.161) | (0.163) | (0.163) |
| Linked Fate (Intra) | 0.331^**^ | 0.240^*^ | 0.212 |
|  | (0.132) | (0.133) | (0.133) |
| Age | -0.153 | -0.351 | -0.350 |
|  | (0.327) | (0.331) | (0.331) |
| Income | 0.313^**^ | 0.188 | 0.214 |
|  | (0.157) | (0.159) | (0.159) |
| Education | 0.878^***^ | 0.701^***^ | 0.677^***^ |
|  | (0.221) | (0.224) | (0.224) |
| Female | 0.025 | 0.080 | 0.081 |
|  | (0.089) | (0.090) | (0.090) |
| Not Born in the U.S. | -0.268^***^ | -0.262^**^ | -0.262^**^ |
|  | (0.103) | (0.103) | (0.103) |
| Civic Org Involvement | -0.171 | -0.199 | -0.181 |
|  | (0.152) | (0.152) | (0.152) |
| Co-ethnic Neighborhood | 0.706^***^ | 0.553^***^ | 0.476^***^ |
|  | (0.120) | (0.122) | (0.124) |
| Strength of Partisanship |  | 0.057 | 0.054 |
|  |  | (0.041) | (0.041) |
| Conservative |  | -0.145 | -0.130 |
|  |  | (0.133) | (0.133) |
| Republican |  | -0.203 | -0.200 |
|  |  | (0.140) | (0.141) |

| Interest in Politics |  | 1.192^***^ | 1.152^***^ |
| --- | --- | --- | --- |
|  |  | (0.188) | (0.189) |
| Internal Efficacy |  | 0.263^*^ | 0.273^*^ |
|  |  | (0.147) | (0.147) |
| External Efficacy |  | -0.314^*^ | -0.282^*^ |
|  |  | (0.167) | (0.167) |
| Recruitment |  |  | 0.312^***^ |
|  |  |  | (0.089) |
| Constant | -2.688^***^ | -3.194^***^ | -3.237^***^ |
|  | (0.220) | (0.252) | (0.253) |
| Observations | 2,388 | 2,388 | 2,388 |
| Log Likelihood | -Inf.000 | -Inf.000 | -Inf.000 |
| Akaike Inf. Crit. | Inf.000 | Inf.000 | Inf.000 |

*Note*: Poisson regression coefficients with standard errors in parentheses. *p=0.1; **p<0.05;

***p=0.01.

# Table F: Inter and Intra-Racial Linked Fate on Asian American Unconventional Political Participation

| *Dependent variable: Unconventional Political Participation* | | | |
| --- | --- | --- | --- |
| Asian Americans Only | | | |
| Minority Linked Fate (Inter) | 0.652^***^ | 0.447^**^ | 0.417^**^ |
|  | (0.197) | (0.194) | (0.195) |
| Linked Fate (Intra) | 0.445^***^ | 0.345^**^ | 0.312^**^ |
|  | (0.151) | (0.150) | (0.151) |
| Age | -0.334 | -0.354 | -0.366 |
|  | (0.339) | (0.340) | (0.339) |
| Income | 0.238 | 0.127 | 0.117 |
|  | (0.172) | (0.174) | (0.174) |
| Education | 0.259 | 0.061 | 0.077 |
|  | (0.259) | (0.262) | (0.263) |
| Female | -0.0001 | 0.072 | 0.097 |
|  | (0.098) | (0.100) | (0.100) |
| Not Born in the U.S. | -0.234^**^ | -0.191^*^ | -0.171^*^ |
|  | (0.098) | (0.099) | (0.099) |
| Civic Org Involvement | -0.231 | -0.138 | -0.130 |
|  | (0.214) | (0.216) | (0.217) |
| Co-ethnic Neighborhood | 1.100^***^ | 0.847^***^ | 0.785^***^ |
|  | (0.126) | (0.132) | (0.134) |
| Strength of Partisanship |  | 0.119^**^ | 0.115^**^ |
|  |  | (0.048) | (0.048) |
| Conservative |  | -0.011 | -0.017 |
|  |  | (0.144) | (0.144) |
| Republican |  | -0.336^**^ | -0.325^**^ |
|  |  | (0.152) | (0.152) |

| Interest in Politics |  | 1.293^***^ | 1.232^***^ |
| --- | --- | --- | --- |
|  |  | (0.215) | (0.215) |
| Internal Efficacy |  | 0.279 | 0.310^*^ |
|  |  | (0.177) | (0.178) |
| External Efficacy |  | -0.352^*^ | -0.347^*^ |
|  |  | (0.195) | (0.195) |
| Recruitment |  |  | 0.301^***^ |
|  |  |  | (0.106) |
| Constant | -2.511^***^ | -3.122^***^ | -3.150^***^ |
|  | (0.259) | (0.296) | (0.295) |
| Observations | 2,266 | 2,266 | 2,266 |
| Log Likelihood | -Inf.000 | -Inf.000 | -Inf.000 |
| Akaike Inf. Crit. | Inf.000 | Inf.000 | Inf.000 |

*Note*: Poisson regression coefficients with standard errors in parentheses. *p=0.1; **p<0.05;

***p=0.01.
